# A Reading Range- and Frequency-Reconfigurable Antenna for Near-Field and Far-Field UHF RFID Applications

**DOI:** 10.3390/s25020408

**Published:** 2025-01-11

**Authors:** Chenyang Song, Zhipeng Wu

**Affiliations:** Department of Electrical and Electronic Engineering, The University of Manchester, Manchester M13 9PL, UK; songchenyang9454@hotmail.com

**Keywords:** reconfigurable antenna, ultrahigh frequency (UHF), radio frequency identification (RFID), near-field antenna, composite right/left-handed material

## Abstract

In radio frequency identification (RFID), differences in spectrum policies and tag misreading in different countries are the two main issues that limit its application. To solve these problems, this article proposes a composite right/left-handed transmission line (CRLH-TL)-based reconfigurable antenna for ultra-high frequency near-field and far-field RFID reader applications. The CRLH-TL is achieved using a periodically capacitive gap-loaded parallel plate line. By deploying the CRLH-TL operating at zeroth-order resonance, a loop antenna with in-phase radiating current is obtained, which contributes to a strong and uniform H-field and a horizontally polarized omnidirectional radiation pattern. By introducing additional tunable components, frequency and reading range reconfigurabilities are enabled. The frequency tuning range is from 833 MHz to 979 MHz, which covers the worldwide UHF RFID band. Moreover, each operation mode has a narrow frequency band, which means it can operate without violating different countries’ radio frequency policy and reduce the design difficulty of designing multiple versions of a reader. Both the near-field interrogation zone and maximum far-field reading distance of the antenna are adjustable. The near-field interrogation zone is 400 mm × 400 mm × 50 mm and can be further confined. The tuning range for far-field reading distance is from 2.71 m to 0.35 m.

## 1. Introduction

Radio frequency identification (RFID) is one of the most widely used technologies in object identification and product management. Recently, RFID has attracted the attention of more researchers because it plays an important role in the underlying layer of internet of things (IoT), such as product track and trace, pervasive sensing, security, and object localization [1,2,3,4]. In addition, the ultra-high-frequency (UHF) RFID is highly valued in practical applications due to its outstanding reading distance, compatibility, and large chip memory. The investigation of UHF RFID reader antenna is valuable in efforts to meet the RF requirements of novel IoT applications.

In the different application scenarios, near-field and far-field RFID reader antennas have been investigated. In near-field applications, the maximum tag detection distance is usually confined to 1 m and the primary task is to enlarge the near-field interrogation zone within this distance. Consequently, strong and uniform E/H-fields are used. Designs such as meandered-line antenna [5], log-periodic antenna [6], patch antenna [7], and slot antenna [8] are developed to provide E-field coupling. Compared with the E-field antennas, H-field antennas are used more widely in near-field RFID. The strong and uniform H-fields are generated with approaches including the use of traveling-wave antennas [9,10], composite right/left-handed (CRLH) material-based antennas [11,12,13], and loop antennas [14,15,16,17]. The recently designed far-field antenna aims to increase the far-field RFID coverage while having the minimum antenna size [18].

However, the development of multi-service RFID has required antennas to have both near-field and far-field radiation capabilities. Moreover, some applications require a switchable reading zone to avoid misreading. Although there are a few investigations on RFID reader antennas operating in both near- and far-fields [19,20,21], the antennas cannot meet the requirement of a confinable reading range. Additionally, different countries defined different operation frequencies for UHF RFID. Multiple versions of RFID readers are thus manufactured, which results in trouble in international trade. Reconfigurable antennas use a technology that involves tuning the antenna’s electrical and propagation performances, which provides a potential solution for improving UHF RFID.

Reconfigurable antennas have become a key focus in modern antenna research due to their adaptability and potential for use in diverse applications. The common techniques for achieving reconfigurability can be broadly categorized into three types: electrical-, mechanical-, and material-based methods. Electrical reconfiguration is the most widely employed technique, involving the use of active components such as PIN diodes, varactor diodes, and RF switches to dynamically alter the antenna’s frequency, polarization, and radiation pattern by controlling the current distribution on the radiating structure [22,23]. The active components offer electric-controlled reconfigurabilities, which are simple, fast and cost-effective. Mechanical reconfiguration, on the other hand, relies on physically altering the geometry of the antenna, such as by rotating parts or adjusting the length of elements, to achieve performance variation [24,25]. This method offers significant reconfigurability but at the cost of slower response times and increased mechanical complexity. Material-based reconfiguration leverages advanced materials like liquid crystals, ferrites, or phase-change materials, whose electromagnetic properties can be modified by external stimuli, enabling seamless the tuning of the antenna’s parameters [26,27]. Each of these approaches has unique strengths and trade-offs, and the choice of method is determined by application-specific requirements such as switching speed, operational bandwidth, and implementation complexity. The utilization of these reconfiguration techniques in antenna design offers substantial benefits in enhancing the functionality of RFID systems and IoT applications.

To provide a technical solution for these challenges, this paper proposes a reading range and frequency-reconfigurable loop antenna based on CRLH transmission line (TL) technology. On the basis of providing a strong and uniform H-field for near-field interrogation and an omnidirectional far-field radiation, the antenna provides a reconfigurable operation frequency covering the worldwide UHF RFID frequency band and a controllable tag interrogation zone.

In the following sections, Section 2 describes the operational principle and configuration of the CRLH-TL-based reconfigurable loop antenna. The simulated and measured results of the proposed antenna are presented and discussed in Section 3. The RFID validation of the fabricated antennas is given in Section 4. In Section 5, conclusions are drawn.

## 2. Operational Principles and Designs

### 2.1. CRLH-TL-Based Zero-Phase-Shifting-Line

The loop antenna is a suitable solution for near-field and far-field RFID applications, with a good performance according to its H-field, and can confine its reading range to be mostly within the main axial lobe. However, the present UHF loop antennas have some defects in input impedance matching and problems in the uniformity of the currents along the loop. Recently, studies of left-handed (LH) metamaterials, which are based on periodic structures, have progressed rapidly. The negative material with permittivity and permeability was first studied theoretically by Veselago in 1968, and is well known as the left-handed (LH) material [28]. CRLH material is a kind of novel material that has special properties that do not exist in natural materials. In short, the most significant and unique property of the CRLH material-based transmission line is that it contains parasitic series capacitance and shunt inductance, which are called the left-hand capacitance and left-hand inductance [28]. Recently, permittivity-negative wires [29], permeability-negative ring resonators [29,30,31], negative-refractive-index (NRI) materials [32], and double-negative materials (DNM) [33,34] have been investigated.

**Figure 1:**
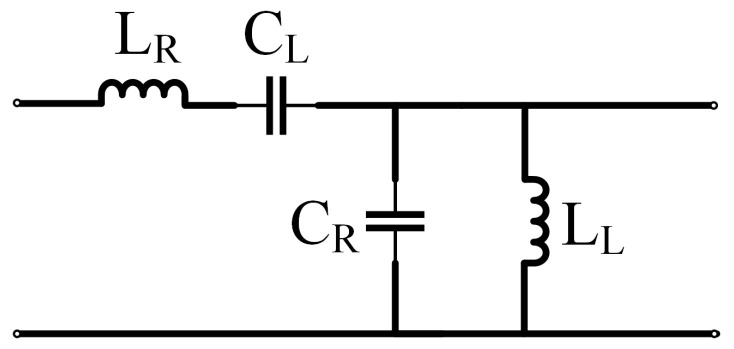
The equivalent circuit of the CRLH transmission line.

The equivalent circuit of the CRLH transmission line model is shown in Figure 1. According to transmission line theory and the CRLH transmission line characteristic [35], the equivalent permittivity and permeability of the CRLH transmission line can be expressed as follows:

(1) $$\mu_{CRLH}=\frac{Z^{\prime }}{j\omega }=L_{R}^{\prime }-\frac{1}{\omega^{2}C_{L}^{\prime }}$$

(2) $$\varepsilon_{CRLH}=\frac{Y^{\prime }}{j\omega }=C_{R}^{\prime }-\frac{1}{\omega^{2}L_{L}^{\prime }}$$

where ω is the angular frequency, and *L*′*_R_* and *C*′*_R_* are the RH inductance and capacitance in unit length. Consequently, the phase constant can be controlled by varying the equivalent permittivity and permeability. The wave in the transmission line remains in phase if the phase constant is adjusted to zero. This technology can be used to generate zero-phase-shift-line (ZPSL) transmission lines and is the entry point of the UHF RFID antenna application. The phenomenon where the phase constant is tuned to zero is called zeroth-order resonance (ZOR). A CRLH-TL theory is hence developed based on the CRLH metamaterial [36]. A homogeneous model of an ideal TL is composed of series inductance and shunt capacitance, which are called the right-handed (RH) inductance and capacitance. For CRLH-TL, left-handed (LH) series capacitance and shunt inductance are introduced, which results in changes in propagation properties. The phase constant of the CRLH-TL can be expressed using the Bloch–Floquet equation [36]:

(3) $$\beta \left(\omega \right)=s\left(\omega \right)\sqrt{\omega^{2}L_{R}^{\prime }C_{R}^{\prime }+\frac{1}{\omega^{2}L_{L}^{\prime }C_{L}^{\prime }}-\left(\frac{L_{R}^{\prime }}{L_{L}^{\prime }}+\frac{C_{R}^{\prime }}{C_{L}^{\prime }}\right)}$$

where *s*(ω) is the sign function, which can be written as

(4) $$s\left(\omega \right)=\left\{\begin{array}{c} -1,\omega <\omega_{\Gamma 1}=min\left(\frac{1}{\sqrt{L_{R}^{\prime }C_{L}^{\prime }}},\frac{1}{\sqrt{L_{L}^{\prime }C_{R}^{\prime }}}\right)\\ +1,\omega >\omega_{\Gamma 2}=max\left(\frac{1}{\sqrt{L_{R}^{\prime }C_{L}^{\prime }}},\frac{1}{\sqrt{L_{L}^{\prime }C_{R}^{\prime }}}\right) \end{array}\right.$$

Consequently, the phase constant is variable for CRLH-TL, which can be utilized to construct zero-phase-shifting-line loop antennas, which provide strong and uniform H-fields by tuning the phase constant to zero.

With the assistance of the CRLH transmission line at ZOR, a loop antenna with a strictly in-phase current distribution can be constructed. In order to reach the ZOR of the CRLH, the LH inductance is not introduced while the LH capacitance is introduced. This type of transmission line is called a permeability-negative transmission line [29]. According to recent research, there are multiple approaches to introducing LH capacitance, such as interdigital structures [37,38,39], segmented loops [40], and lumped elements [41]. In this article, a parallel plate line-based permeability-negative transmission line is used to construct a ZPSL loop antenna, with frequency and gain reconfigurability, for UHF RFID applications.

### 2.2. Capacitive Gap-Loaded Parallel Plate Line

**Figure 2:**
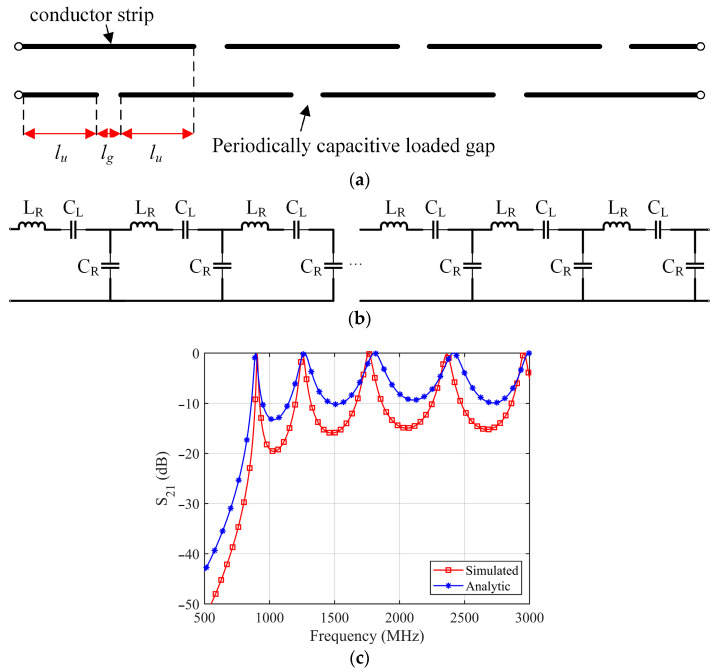
(**a**) Periodically capacitive gap-loaded parallel plate line; (**b**) equivalent circuit of capacitive-loaded parallel plate line; (**c**) simulated and analyzed S_21_.

To achieve the ZPSL structure, a periodically capacitive gap-loaded parallel plate line is proposed. Figure 2a shows the gap-loaded parallel plate line. The parallel plate line is composed of two parallel conductor strips separated by a dielectric substrate. The conductor strips on the dielectric are alternately slotted every *l_u_* distance. By repeating the operation, the parallel plate line forms a periodically capacitive gap-loaded parallel plate line. By introducing the gaps, only LH or series capacitance is added to the parallel plate line [42]. The equivalent circuit is shown in Figure 2b. Therefore, the Equation (3) can be rewritten as follows:

(5) $$\beta \left(\omega \right)=s\left(\omega \right)\sqrt{\omega^{2}L_{R}^{\prime }C_{R}^{\prime }-\frac{C_{R}^{\prime }}{C_{L}^{\prime }}}$$

The phase constant is configured to zero when achieving the ZOR. The resonant frequency at ZOR is written as follows:

(6) $$f_{ZOR}=\frac{\omega }{2\pi }=\frac{1}{2\pi \sqrt{L_{R}C_{L}}}$$

At ZOR, the radiating current remains in-phase in the TL, which may contribute to the formation of a strong and uniform H-field distribution. For the purpose of validating the equivalent circuit in Figure 2b, a gap-loaded parallel plate line with eight periodically elements is simulated with CST electromagnetic simulation software. The length of each element *l_u_* = 12.5 mm. The width of parallel plate line *w* = 8 mm. The width of te loaded gap *g* = 3 mm. The parallel plate line is designed on a 1.58 mm thick FR-4 substrate (ε_r_ = 4.4, tanδ = 0.020). The values of right-hand lumped elements in the equivalent circuits can be calculated using the parasitic parameter equation of the parallel plate line [43], while the value of left-hand lumped elements can be calculated using the parameter extraction technique [44]. The simulated and analyzed S_21_ values of the proposed model are compared in Figure 2c. According to the results, the full-wave results that we simulated and analyzed agree well. The right-hand capacitance C_R_ = 3.1 pF, right-hand inductance L_R_ = 3.9 nF, and left-hand capacitance C_L_ = 9.5 pF.

### 2.3. ZPSL Loop Antenna

**Figure 3:**
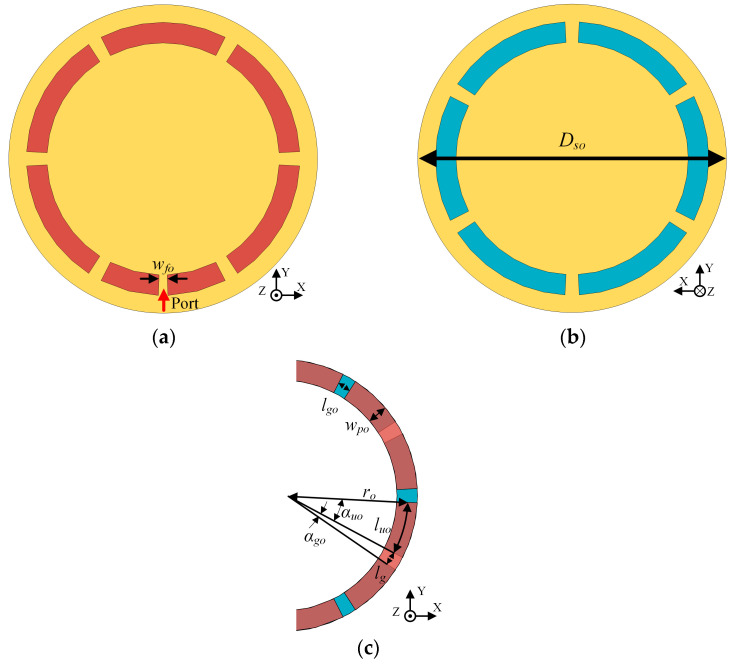
The proposed CRLH-TL loop antenna: (**a**) top view; (**b**) bottom view; (**c**) single unit.

**Figure 4:**
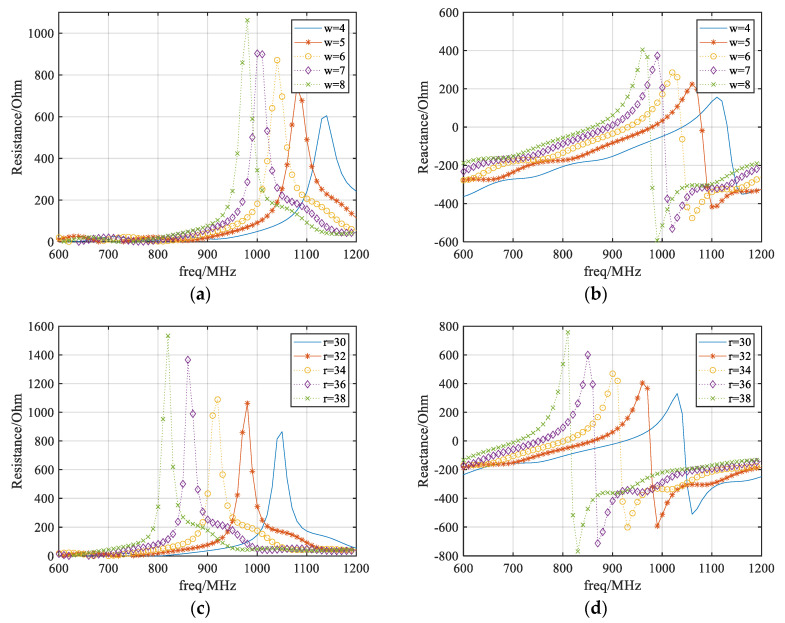

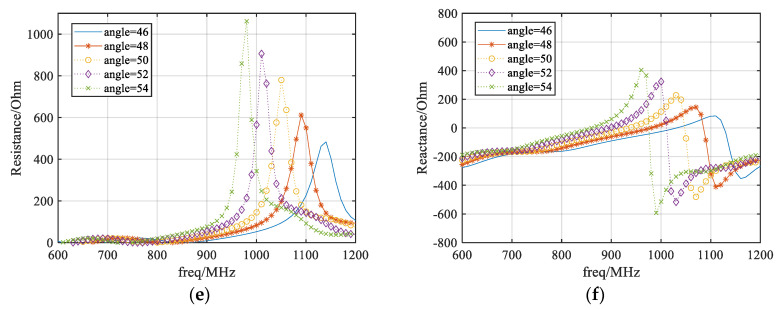
The impedance diagram of the ZPSL loop antenna: (**a**) resistance with varying width; (**b**) reactance with varying width; (**c**) resistance with varying radius; (**d**) reactance with varying radius; (**e**) resistance with varying arc angle; (**f**) reactance with varying arc angle.

Based on the operation principle of CRLH-TL and the gap-loaded parallel plate structure, a ZPSL transmission line loop antenna is designed by looping the gap-loaded parallel plate line. As shown in Figure 3, the antenna is fed through the gap slotted at the middle of one parallel plate element. According to the previous theory, the antenna has several adjustable key parameters relating to width and length of the strip elements. The effects of varying the width and length of the strip elements are investigated based on full-wave electromagnetic simulations using CST Studio Suite 2024. The results are shown in Figure 4. According to the results of the parametric study, the impedance of the ZPSL loop antenna is tuned to 50 Ω. A 12-element CRLH loop antenna is designed on a 1.57 mm thick FR-4 substrate (εr = 4.4, tanδ = 0.020). The detailed dimensions of the loop antenna are shown in Table 1.

**Figure 5:**
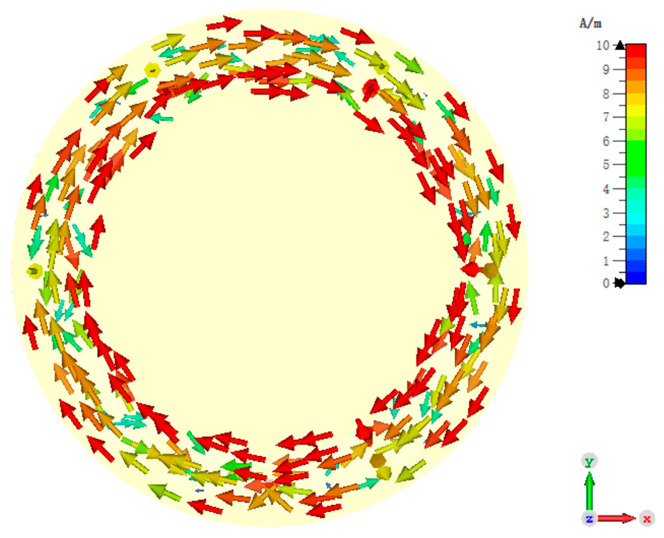
Simulated surface current of the designed CRLH-TL loop antenna at ZOR.

The ZOR frequency is tuned to 866.5 MHz, which is the center frequency of the UK UHF RFID band. The simulated surface current distribution of the ZPSL transmission line loop antenna at 866.5 MHz is shown in Figure 5. According to the simulated surface current distribution, the loop antenna achieves ZOR at 866.5 MHz. The phase constant is zero and the surface current remains in phase, which results in strong and uniform results in its near-field distribution. The near-field distribution can benefit the near-field RFID tag interrogation, which results in its good performances in applications such as smart shelves, asset management, and RFID tag-based sensing. 

**Figure 6:**
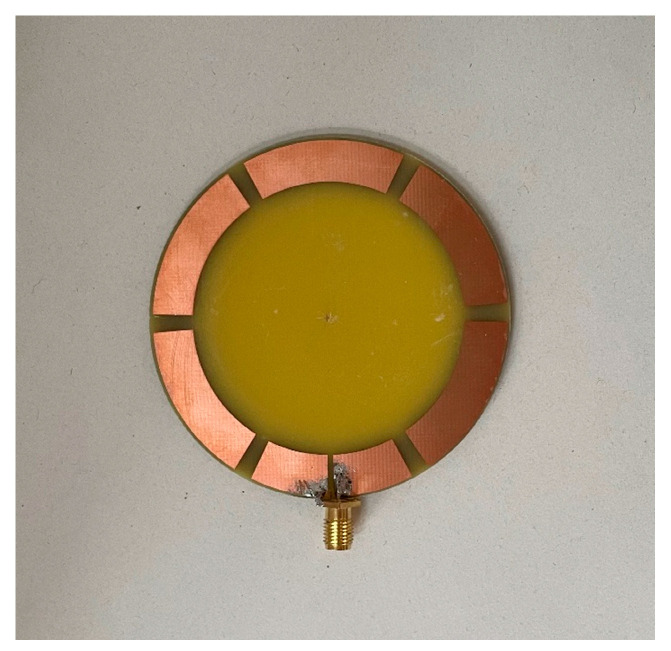
Fabricated CRLH-TL antenna.

**Figure 7:**
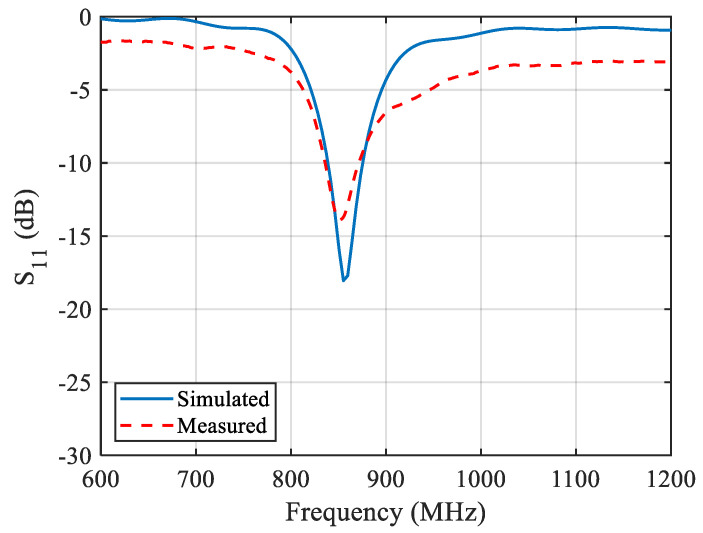
Simulated and measured S_11_ values of the proposed CRLH-TL loop antenna.

**Figure 8:**
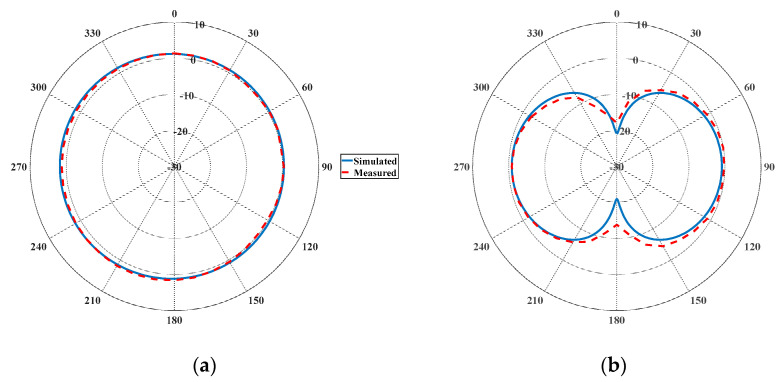
Simulated and measured far-field gain of the proposed CRLH-TL loop antenna: (**a**) E-plane; (**b**) H-plane.

The prototyped ZPSL loop antenna is fabricated on a 1.57 mm thick FR-4 substrate (εr = 4.4, tanδ = 0.020), which is shown in Figure 6. The loop antenna is measured with the Agilent E5071 programmable VNA. The simulations and measurements are shown in Figure 7. The loop antenna is measured to have an impedance bandwidth (S11 < −10 dB) of 35.37 MHz (4.08%, 836.55 MHz–871.92 MHz), which can cover the entire UK UHF RFID band. The simulated S_11_ agrees well with the measurements. In order to evaluate the propagation properties of the prototyped antenna, the radiation pattern of the antenna is measured in an anechoic chamber. The simulated and measured radiation patterns are depicted in Figure 8. The antenna achieves a horizontally polarized omnidirectional radiation pattern with 1.6 dB peak gain with a gain variation of only 1.2 dB on the H-plane. The half-power beam width (HPBW) is 84 degrees. The far-field propagation properties of the antenna can enlarge the far-field tag interrogation zone of RFID readers in applications such as indoor localization, entry control, and asset management.

Overall, the ZPSL loop antenna provided a compact antenna solution with an enlarged near- and far-field interrogation zone for RFID applications. However, simply enlarging the tag interrogation zone cannot fulfill the needs of most of the modern RFID applications. In different countries, UHF RFID shares different frequency bands, such as 865–868 MHz in Europe, 902–928 MHz in US, 840–845 and 920–925 MHz in China. This has caused additional costs in developing hardware and additional operations in cross-border applications. Moreover, in some applications, the targeted detection zone is tunable. For example, the misreading in far-field operations is not acceptable in near-field-only applications. Therefore, a reconfigurable loop antenna is developed in this article based on the CRLH-based ZPSL loop antenna. 

### 2.4. Reconfigurable CRLH-TL Loop Antenna

**Figure 9:**
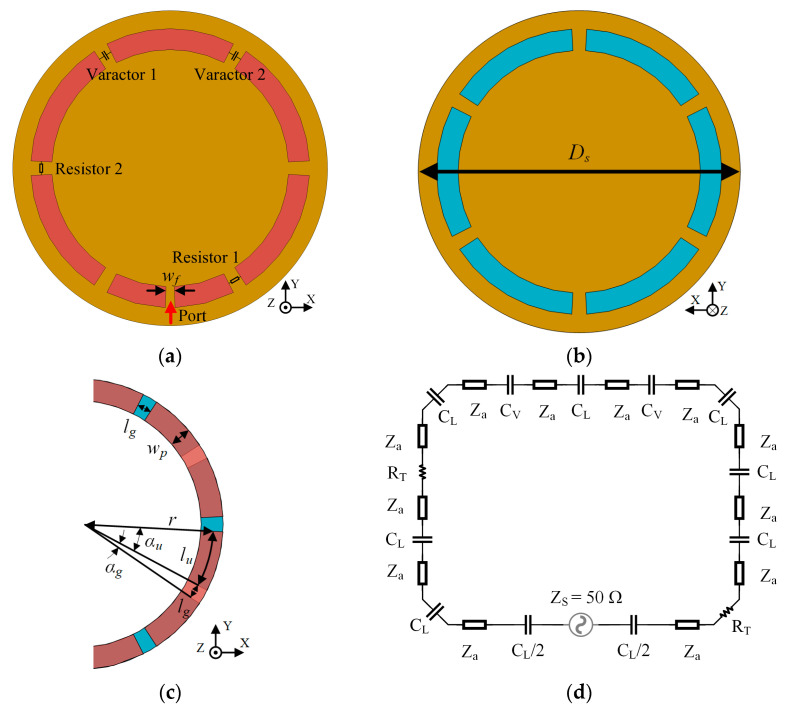
The proposed reconfigurable CRLH-TL loop antenna: (**a**) top view; (**b**) bottom view; (**c**) single unit; (**d**) equivalent circuit.

The designed prototype of the CRLH-TL loop antenna is shown in Figure 9. The parallel plate line lies on a 1.57 mm thick circular FR-4 substrate (ε_r_ = 4.4, tanδ = 0.020) and is slotted periodically to introduce LH capacitance. The antenna is designed to have 12 periodical units, each containing a unit of parallel plate line and a loaded gap, which is shown in Figure 9c. The antenna is fed at the center of one strip section. To achieve frequency reconfigurability, varactors are added in addition to the loaded gaps, which provides tunable LH capacitance. Two of varactors with a variation range from 2.1 pF to 18.7 pF are added to the two gaps furthest from the feed point to enlarge frequency tuning range to cover the worldwide UHF RFID frequency. Moreover, trimmer resistors are added to adjust the intensity of the radiating current. Figure 9d shows the equivalent circuit of the loop antenna. In this way, the far-field gain and H-field strength are controlled, corresponding to a reconfigurable far-field reading distance and a near-field interrogation zone. The dimensions and the position of trimmer resistors are optimized with the CST full-wave simulation to make the antenna match to 50 Ohm in every operation mode. Table 2 shows the optimized dimensions of the antenna.

## 3. Near- and Far-Field Simulation and Measurements of the Reconfigurable Antenna

**Figure 10:**
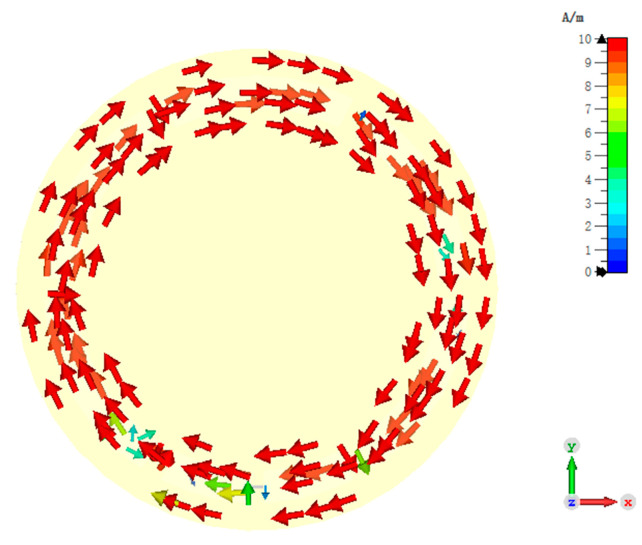
Surface current distribution of the proposed reconfigurable antenna.

**Figure 11:**
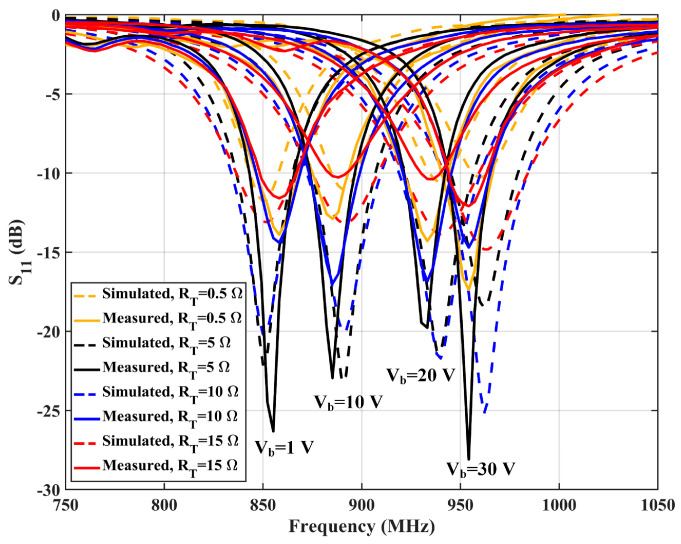
Simulated and measured S11 of the proposed reconfigurable antenna in different operation modes.

To validate the performance of the design, the antenna is simulated with the CST full-wave electromagnetic simulation software and measured with an Agilent E5071 vector network analyzer (Agilent Technologies, Manchester, UK). The bias voltage varies from 1 V to 30 V to provide between 2.1 pF and 18.7 pF capacitance. The trimmer resistors are tuned from 0.5 Ohm to 15 Ohm to vary the reading distance. Figure 10 shows the simulated surface current of the antenna at ZOR. An in-phase surface current is achieved. As illustrated in Figure 10, the antenna shows an in-phase surface current distribution, which results in a strong vertical H-field. In addition, the current intensity is not strictly uniform because of the introduction of the trimmer resistors and the varactors. This may cause distortion in the H-field distribution and the far-field radiation pattern, which will be analyzed later. Figure 11 shows the simulated and measured S11 of the proposed antenna. According to the results, the designed antenna has an impedance bandwidth (S11 < 10 dB) of 23.78 MHz (2.74%, 846.43–870.21 MHz) for the 866.5 MHz operating mode. This narrow single-mode impedance bandwidth avoids violating national RFID radio frequency (RF) regulations, even if a wideband RFID reader is used. The overall impedance bandwidth is 146.72 MHz (16.93%, 832.50–979.22 MHz) when the trimmer resistors are set to 0.5 Ohm, which can cover the worldwide UHF RFID band.

**Figure 12:**
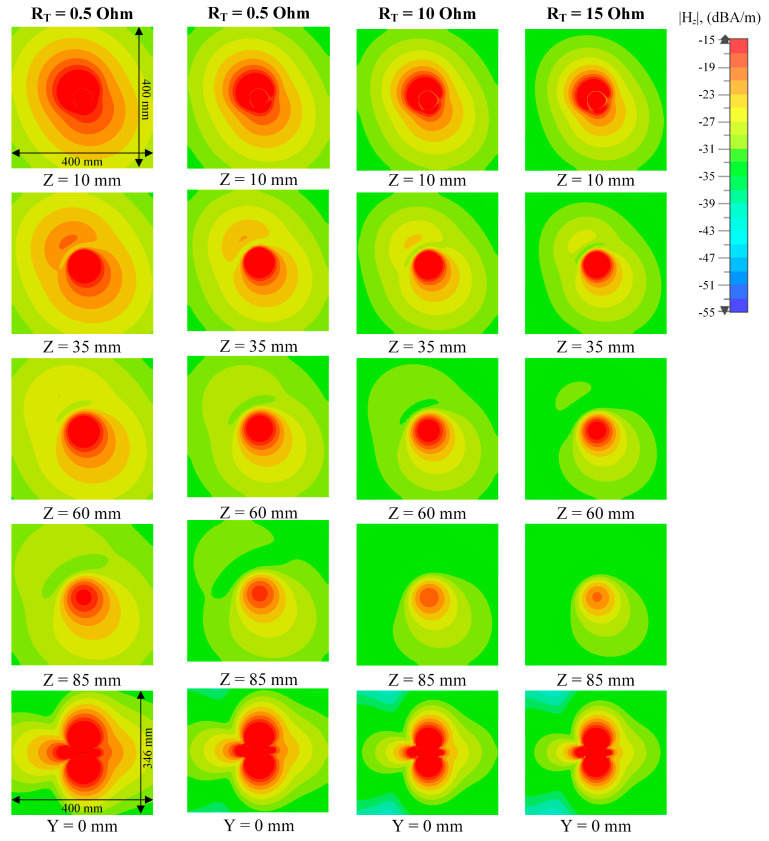
Simulated vertical H-field (|H_Z_|) distributions on the X-Y plane and X-Z plane at different reading distances of the proposed reconfigurable antenna.

**Figure 13:**
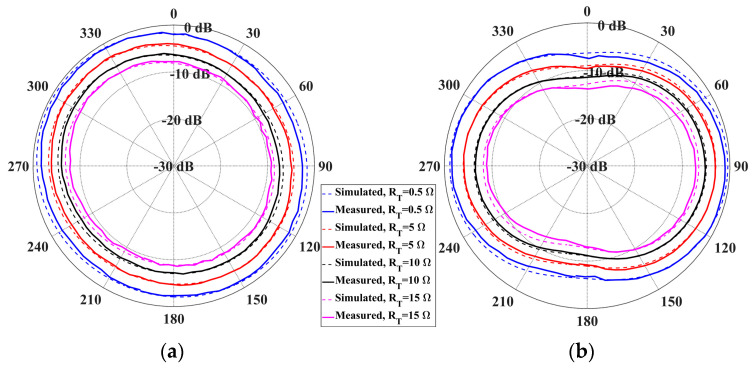
Simulated and measured far-field gain of the reconfigurable antenna at 866.5 MHz: (**a**) E-plane; (**b**) H-plane.

The simulated vertical H-field distributions on X-Y planes at differing reading height Z are shown in Figure 12. A vertical H-field stronger than 0.02 A/m is generated over an area of 400 mm × 400 mm within 50 mm distance when trimmer resistors read at 0.5 Ohm, which is sufficient for RFID operation. The H-field is suppressed when the resistance of the trimmer resistor is increased, which results in a confined near-field detection area and a reduced tag reading distance. The far-field radiation patterns at an 866.5 MHz operating frequency with different tuning resistances are shown in Figure 13. The antenna is measured to have a maximum far-field gain of −0.6 dB with a 1.9 dB gain variation when operating in the multi-service mode (R = 0.5 Ohm). The distortion caused by the trimmer resistors and varactors are acceptable. The maximum far-field gain is suppressed to −7.1 dB when the resistance is increased to 15 Ohm, which can be considered as a pure-near-field mode. The practical impact of resistance on the interrogation zone is measured.

## 4. RFID Validation and Performance Comparison

**Figure 14:**
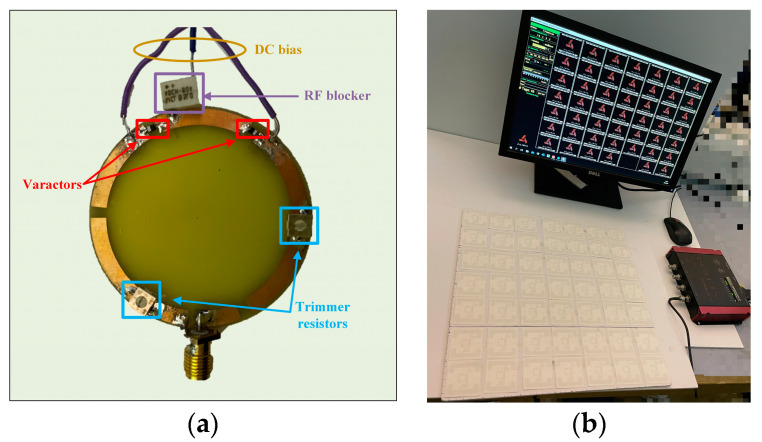
(**a**) Prototyped reconfigurable CRLH-TL loop antenna; (**b**) experiment setup.

**Figure 15:**
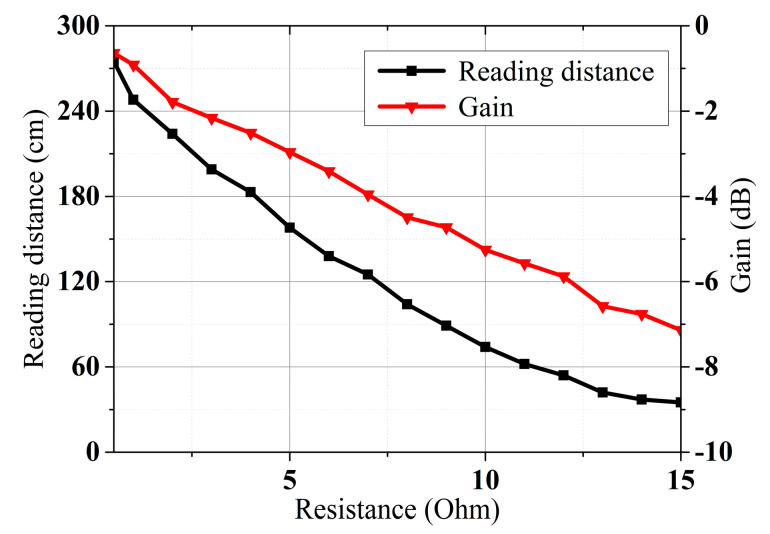
Gain and maximum reading distance of the designed reconfigurable antenna.

**Figure 16:**
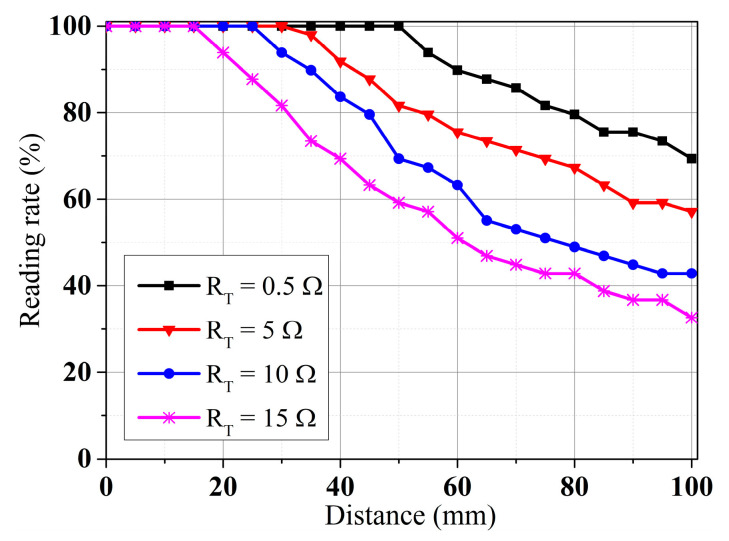
Near-field tag detection rate of the proposed reconfigurable antenna.

**Figure 17:**
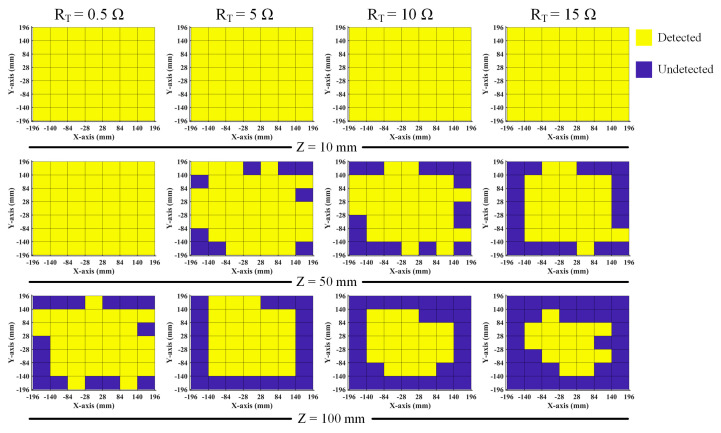
Near-field tag detection zone at different reading heights.

To validate the antenna’s RFID performance in the different operating modes, the CRLH-TL loop antenna is validated with tag detection tests. Figure 14a shows the prototyped antenna. An RF blocker is used to filter the impact from external DC bias voltage. Figure 14b shows the experimental setup. The tag detection test is performed with an Alien 9900+ RFID reader (Alien Technology, Manchester, UK). A 400 mm × 400 mm foam board is divided equally into 49 cells, each attaching an ALN-9634 tag to test the NF interrogation zone of the loop antenna [45]. The tag board is placed above the antenna and is gradually moved away from the loop antenna in 10 mm increments. Also, the maximum far-field tag reading distance of the antenna is recorded. Figure 15 shows the trends of gain and far-field reading distance when the tuning resistance is increased. The maximum far-field reading distance is decreased to 35 cm from 272 cm while the peak far-field gain is suppressed to −7.1 dB from −0.6 dB, which changes the antenna to near-field mode from multi-service mode. Figure 16 shows near-field tag reading rate in the near-field test and Figure 17 shows the corresponding reading cells of the tag board at different reading distances. The R = 0.5 Ohm mode provides a reading zone of 400 mm × 400 mm × 50 mm or a higher reading distance in the central area, while the R = 15 Ohm mode provides a reading zone of 400 mm × 400 mm × 20 mm. Moreover, the reading zone is confined in higher reading heights when the resistance is increased. Table 3 shows the performance comparison of this work and state-of-art investigations. Compared with other works, this design has overcome shortcomings such as low interrogation distance [17,46], and narrow bandwidths [5,8,17]. Compared with the existing loop antenna such as [14,17] and CRLH structures such as [14,39], this work has lower design complexity, a miniature size, and reconfigurability.

Also, each operation mode has a narrow frequency band to avoid violating RF regulations. A large interrogation zone is generated with a relatively small antenna size in this work. Moreover, the proposed loop antenna provides muti-service RFID. Also, this antenna is the first solution to support an adjustable frequency and reading range.

## 5. Conclusions

A CRLH-TL-based frequency and reading range reconfigurable loop antenna for near- and far-field RFID applications is proposed in this paper. The antenna achieves an in-phase loop radiating current with the gap-loaded parallel plate line and enables dual reconfigurability with additional trimmer resistors and varactors. The adjustable frequency band covers the worldwide UHF RFID band and is valued in international trade applications. The antenna provides a near-field RFID interrogation zone of 400 mm × 400 mm × 50 mm in the multi-service mode and can be further confined. The antenna also provides an omnidirectional far-field radiation, which results in far-field tag reading distances ranging from 0.35 m to 2.71 m. The tunable reading ranges can limit the detection distance and can avoid misreading, which are applicable for multi-service RFID scenarios such as proximity detection, object detection, and localization. 

## Figures and Tables

**Table 1:** Detailed dimensions of CRLH-TL loop antenna.

Parameter	Description	Value
*D_so_*	Diameter of the antenna	64.5 mm
*r* *_o_*	Radius of the CRLH TL loop	30 mm
*w_p_* *_o_*	Width of parallel plate line	8 mm
*w_fo_*	Width of feed slot	1.5 mm
*l_uo_*	Length of single plate line	12.57 mm
*l_go_*	Gap length between plate lines	3.14 mm
*α_uo_*	Degree of each unit	24°
*α_go_*	Degree of each gap	6°
*N_o_*	Number of parallel plate units	12

**Table 2:** Detailed dimensions of the reconfigurable CRLH-TL loop antenna.

Parameter	Description	Value
*D_s_*	Diameter of the antenna	52.5 mm
*r*	Radius of the CRLH TL loop	21.5 mm
*w_p_*	Width of parallel plate line	3.5 mm
*w_f_*	Width of feed slot	1.5 mm
*l_u_*	Length of single plate line	9.0 mm
*l_g_*	Gap length between plate lines	2.25 mm
*α_u_*	Degree of each unit	24°
*α_g_*	Degree of each gap	6°
*N*	Number of parallel plate units	12

**Table 3:** Performance comparison of state-of-the-art investigations of UHF RFID reader antennas.

Ref.	Methods	Bandwidth (MHz)	Purpose Field	Antenna Size (mm^2^)	Reading Area (mm^2^)	Reading Distance		FF Radiation	Gain (dBi)	Output Power (dBm)	Reconfigurability
						NF (mm)	FF (m)				
[5]	Meander line	917–937 (2.17%)	NF	270 × 150	400 × 320	300	n.a.	n.a.	−6	30	n.a.
[8]	Meander line	896–946 (5.43%)	NF & FF	300 × 300	300 × 300	n.a.	0.6	Directional	3.3	31.5	n.a.
[14]	Loop array	896–952 (6.06%)	NF	173.5 × 173.5	radius = 80	120	n.a.	Directional	n.a.	30	n.a.
[17]	Loop array	913–932 (2.07%)	NF	289 × 240	270 × 240	43	n.a.	n.a.	−22.5	30	n.a.
[47]	CRLH material	869–936 (7.40%)	FF	75 × 75	n.a.	n.a.	9.3	Directional	5.72	n.a.	n.a.
[46]	Dipole array	870–930 (6.65%)	NF or FF	280 × 280	300 × 300	25.	n.a.	Directional	7	27	Switchable detection zone
This work	CRLH loop	833–979 (16.93%)	NF & FF	53 × 53	400 × 400	0.5 Ω mode: 50	2.72	Omnidirectional	−0.6	30	Frequency & detection zone

## Data Availability

The raw data supporting the conclusions of this article will be made available by the authors on reasonable request.
